# Circulating adiponectin levels, expression of adiponectin receptors, and methylation of adiponectin gene promoter in relation to Alzheimer’s disease

**DOI:** 10.1186/s12920-022-01420-8

**Published:** 2022-12-16

**Authors:** Aiym Kaiyrlykyzy, Bauyrzhan Umbayev, Abdul-Razak Masoud, Aida Baibulatova, Andrey Tsoy, Farkhad Olzhayev, Dinara Alzhanova, Gulnaz Zholdasbekova, Kairat Davletov, Ainur Akilzhanova, Sholpan Askarova

**Affiliations:** 1 Center for Life Sciences, National Laboratory Astana, Nazarbayev University, 53 Kabanbay Batyr Avenue, Astana, Kazakhstan; 2 Faculty of Medicine and Public Health, al-Farabi Kazakh National University, Almaty, Kazakhstan; 3 Louisiana Tech University, Ruston, LA USA; 4 Department of Neurology and Psychiatry, Astana Medical University, Astana, Kazakhstan; 5 Open Clinic, Astana, Kazakhstan; 6 Karaganda State Medical University, Karaganda, Kazakhstan; 7 Asfendiyarov Kazakh National Medical University, Almaty, Kazakhstan

**Keywords:** Alzheimer’s disease, Adiponectin, AdipoR1, AdipoR2, DNA methylation

## Abstract

**Background:**

The role of adiponectin (ADIPOQ) in Alzheimer’s disease (AD) has been documented, however, demonstrating controversial results. In this study, we investigated blood serum ADIPOQ levels, methylation of the adiponectin gene promoter, and adiponectin receptors (AdipoR1 and AdipoR2) expression in blood samples isolated from AD patients and healthy controls.

**Methods:**

We performed a case–control study including 248 subjects (98 AD patients and 150 healthy controls); ADIPOQ serum levels, AdipoR1, and AdipoR2 levels in PBMC were measured by ELISA Kits, and ADIPOQ gene methylation was analyzed using methyl-specific PCR.

**Results:**

Serum adiponectin levels were threefold higher in the AD group compared to the controls. We have also found a positive correlation between adiponectin and MMSE scores and high-density lipoprotein cholesterol (HDL-C) in AD patients. A significant difference in the proportion of methylation of the CpG sites at − 74 nt of the ADIPOQ gene promoter was detected in AD cases, and the levels of adiponectin in blood serum were significantly higher in methylated samples in the AD group compared to controls. The amount of AdipoR1 was significantly higher among AD subjects, while the expression of AdipoR2 did not vary between AD patients and controls.

**Conclusion:**

These findings may contribute to a deeper understanding of the etiological factors leading to the development of dementia and may serve as a basis for the development of predictive biomarkers of AD.

## Introduction

Alzheimer's disease (AD) is one of the fastest-growing pathologies globally that affects millions of people, regardless of gender, education, or social origin. It is the most frequent age-related cognitive disorder that accounts for up to 80 percent of all dementia cases [1]. AD may develop in the elderly population due to various factors, ranging from lifestyle, stress levels, genetic factors, and chronic diseases (e.g., cardiovascular and metabolic diseases) to physiological changes in the human body associated with aging. On account of the long clinical course, limited diagnosis, and lack of effective treatment methods, AD is a severe public health problem with enormous socio-economic costs [6].

An increasing body of evidence indicates that AD patients develop peripheral metabolic dysfunctions, which may play a pivotal role in the pathogenesis of AD [2, 3]. In support of this concept, many metabolic disorders, such as obesity, dyslipidemia, insulin resistance, and type 2 diabetes, are the risk factors for AD [2, 3]. Recently, considerable information has been accumulated on the action of adiponectin ADIPOQ, a protein hormone primarily expressed in white adipose tissue. This hormone comprises about 0.01% of the total plasma proteins and regulates insulin sensitivity, glucose homeostasis, catabolism of fatty acids, and anti-inflammatory system through multiple mechanisms [4, 5]. ADIPOQ is involved in the pathogenesis of several age-related diseases, such as atherosclerosis, type 2 diabetes, and cardiovascular disorders [4–10]. Moreover, evidence is beginning to accumulate that adiponectin might be an independent risk factor for AD [11–17].

Several studies found that patients with moderate cognitive impairment (MCI) and Alzheimer's disease (AD) have higher levels of ADIPOQ in their plasma than healthy people [11, 12]. Besides, a case–control study that included AD patients and healthy control subjects revealed a relationship between ADIPOQ gene polymorphism and late-onset AD [13]. Van Himbergen et al. showed that elevated ADIPOQ levels in blood were associated with an increased risk of all types of dementia in women [14]. These data are engaging in light of the fact that women have higher levels of ADIPOQ in their blood plasma and higher prevalence and severity of AD [15]. Some investigations, on the other hand, found no significant changes in plasma adiponectin in AD patients [16] or even found lower plasma levels of ADIPOQ in MCI and AD patients compared to controls [17]. It has also been reported that in patients with AD and MCI, an elevated level of ADIPOQ in the cerebrospinal fluid is associated with smaller depositions of beta-amyloid and a larger volume of the hippocampus [17], and the chronic deficiency of ADIPOQ results in the development of Alzheimer’s type cognitive impairment in old mice [18]. In some studies, adiponectin exerted protective properties against oxidative stress in amyloid-beta-induced neurotoxicity [19]. These controversial data indicate that the role of adiponectin in the etiology of AD is not fully understood, and more studies are needed to reveal an association between adiponectin regulation and AD.

In turn, the biological activity of ADIPOQ depends on the expression of adiponectin receptors. ADIPOQ binds to adiponectin receptor type 1 and type 2 (AdipoRs), and T-cadherin. Changes in the expression of AdipoR1 and AdipoR2 were described in monocytes of type 2 diabetes patients and overweight patients with coronary artery disease [9, 10]. These studies suggested that the downregulation or altered function of AdipoR1 and AdipoR2 might be responsible for the development of the alterations in peripheral tissues. A number of studies have demonstrated that SNPs of ADIPOR1and ADIPOR1 are also associated with type 2 diabetes, metabolic syndrome, and coronary artery disease [20–22]. However, there is no information about the expression levels of adiponectin receptors in relation to AD.

In the past, adiponectin was traditionally considered as an adipokine mainly produced in adipose tissue. However, it has now been established that different cells can synthesize adiponectin [23–25]. There are data showing that adiponectin is expressed in a population of regulatory T-cells residing within the thymic nurse cell complexes [26], in peripheral blood mononuclear cells [27], and in macrophages [24, 28]. Moreover, it has been demonstrated that the anti-inflammatory and insulin-sensitizing effects of ADIPOQ are partially mediated via monocytes [9, 22, 29, 30]. Ott et al. demonstrated that gestational diabetes mellitus was associated with alterations of ADIPOQ mRNA expression in adipose tissues and blood cells. The authors also found that in parallel to reduced mRNA expression, small yet significant alterations in locus-specific DNA methylation of the ADIPOQ gene both in maternal fat and blood cells were associated with gestational diabetes and neonatal outcome as well. [31]. In addition, the report of Houde et al. indicated that DNA methylation patterns of the adiponectin gene are similar between different tissues, including blood [32].

Among the identified binding sites interacting with the positive and negative regulators of the ADIPOQ gene, three promoter elements termed the enhancer boxes (E-boxes) are present in the proximal human adiponectin gene promoter region [33, 34]. Even though E-Box is broadly distributed and plays a potentially important role in transcriptional control mechanisms [35], little attention was paid to the effect of E-boxes with respect to the transcriptional regulation of the human ADIPOQ gene. It was established that class I basic helix-loop-helix (bHLH) protein – E47 and inhibitor of differentiation 3 (Id3) inhibits adiponectin expression via E-boxes of mouse adiponectin gene [36]. However, E-boxes regulate the expression of adiponectin in mouse cells via different proteins than in human cells, and until recently, only one published report regarding Snail as E-box binding transcription regulatory factor in human cells has been available [37]. On the other hand, E-boxes of the proximal region of the adiponectin promoter contain cytosine-phosphate-guanine (CpG) dinucleotide, and DNA methylation can be an alternative pathway for transcriptional control mechanisms of adiponectin expression. In this regard, the CpG site of the E-box motif located at − 74 nt of the ADIPOQ gene promoter in peripheral blood has been studied by García-Cardona et al. [33]. They demonstrated that the methylation frequency of the CpG site at − 74 nt of the ADIPOQ gene promoter was reduced in obese and severely obese teenagers compared to thin or overweight adolescents. Since obesity is one of the crucial contributing factors to AD, we also focused on the CpG site (− 74 nt).

Therefore, in this study, we have investigated the circulating blood serum adiponectin levels, methylation of the CpG sites at − 74 nt of the ADIPOQ gene promoter, and expression of adiponectin receptors (AdipoR1 and AdipoR2) in peripheral blood mononuclear cells (PBMC) isolated from patients with AD in comparison with gender and age-matched healthy individuals since it may serve as a basis for the development of predictive blood-based biomarker of AD.

## Materials and methods

### Research design

The case–control study was carried out on a total of 248 samples, including 150 cognitively normal controls and 98 individuals with probable dementia due to Alzheimer's disease (mild and moderate stages). Selection of the cases has been carried out by the neurology department of the city hospital no.1 in Astana, Kazakhstan. At their initial examination, all subjects aged 60 or older were eligible for this study in order to lower the number of AD patients with a genetic cause. A certified neurologist conducted a systematic clinical interview with patients and a caregiver at the start of the study, which provided a full explanation of the complaints with an emphasis on the onset and early symptoms, medical and family histories, as well as drug histories. To rule out non-disease Alzheimer's causes of cognitive impairment, a comprehensive neurological examination was undertaken. The diagnosis was performed according to the Diagnostic and Statistical Manual of Mental Disorders criteria [38], the National Institute of Neurological and Communicative Disorders, and Stroke–Alzheimer’s Disease and Related Disorders (NINCDS–ADRDA) [39].

For inclusion into the control group, cognitively healthy age, gender, and ethnicity-matched health individuals have been recruited from the outpatient department of the hospital. Controls had a negative self-reported medical history of dementia, cognitive impairment, or memory complaints. The study protocol was approved by the IRB of Nazarbayev University; all recruited subjects or their caregivers provided written informed consent. Fasting blood samples were taken from all study participants for biochemical measurements and the determination of serum adiponectin levels. Immediately after collection, serum samples were examined for routine biochemical parameters, and aliquots of the samples were kept at − 20 °C for further adiponectin assays.

### Immunoenzymatic analysis of adiponectin in serum

The content of adiponectin in the samples was measured by ELISA using a kit for the quantitative determination of serum adiponectin in plasma and cell cultures (Sigma-Aldrich, cat.no: RAB0005) according to the manufacturer's protocol.

### DNA isolation

Qiagen blood kit (QIAamp DNA micro kit cat.no/ID: 56304, Qiagen, Germany) was used to isolate genomic DNA from peripheral blood according to the manufacturer's protocol. DNA integrity and concentration were assessed by agarose gel electrophoresis and spectrophotometric analysis.

### Bisulfite modification of DNA

The DNA was processed using the EpiJET bisulfite conversion kit (Thermo Scientific, cat.no: K1461). Briefly, 2 μg of DNA was added to the mixture containing 85 μl of sodium bisulfite, 15 μl of DNA buffer, and 140 µl of protected RNase water. The bisulfite modification of DNA was carried out in a thermal cycler as follows: 5 min at 95 °C, 25 min at 60 °C, 5 min at 95 °C, 85 min at 60 °C, 5 min at 95 °C, 175 min at 60 °C, and, for the final stage, at 20 °C. For purification, 560 μl BL buffer containing 10 μg/ml "carrier" RNA was added to the DNA samples. After washing, 20 μl of EB buffer was added, and then the DNA was precipitated by centrifugation at 15,000×*g* for 5 min. The modified DNA was stored at − 20 °C.

### Methylation-specific PCR

To profile the methylation of the ADIPOQ gene, the CpG site located at the − 74 nt sequence of the E-box was examined using Methylation-Specific PCR and restriction analysis. The choice of CpG site (− 74nt) in the promoter of the adiponectin gene is based on a study by García-Cardona et al. [33]. Amplification of Bisulfite-Treated DNA was performed on Mastercycler instrument (Eppendorf) using PCR Master Mix (cat.no:4351376, Sigma-Aldrich) in 50 µL of RNAse-free water containing 0.4 µM primers (Table 1, SibEnzyme LLC, Novosibirsk). The following PCR conditions were used: 4 min of initial denaturation at 95 °C, 1 min of denaturation at 94 °C, 15 s of annealing at 53 °C, and 1 min of extension at 72 °C. The PCR cycle was repeated 40 times with a final extension at 72 °C for 10 min, followed by cooling to 4 °C.

**Table 1:** A list of primers

#	Primer name	Primer sequence 5′ –» 3′	Gene	PCR product
1	− 74nt—F	TGCCCCATCTTCTGTTGCTG	− 74nt ADIPOQ	186 base pairs
2	− 74nt—R	AACTCGATGAGGGCCAGAGG		
3	Beta-globin—F	CCACTTCATCCACGTTCACC	Beta-globin	268 base pairs
4	Beta-globin—R	GAAGAGCCTAGGACAGGTAC		

### Restriction analysis

HspA I restriction enzyme (20 units) (cat.no: E069, SibEnzyme LLC, Novosibirsk) was added to the wells containing 50 µl of native DNA mixture (50 µg/ml) in restriction buffer followed by incubation for three hours at 37 °C to hydrolyze unmethylated sites. Methyl-sensitive PCR was performed using 30–40 ng of hydrolyzed, non-hydrolyzed DNA and appropriate primers (Table 1). Primers were selected so that the amplified fragment contained at least one but no more than nine recognition sites for HspA I. Additionally, primers were designed to amplify the beta-globin gene fragment to serve as internal DNA quality control.

Amplification of the beta-globin gene was performed in the same conditions as described previously: a 20 µL reaction mix was used, 4 min of initial denaturation at 95 °C, 1 min of denaturation at 94 °C, 15 s of annealing at 53 °C, 1 min of extension at 72 °C. The PCR cycle was repeated 40 times with a final extension at 72 °C for 10 min. This was followed by cooling to 4 °C.

Separation and visualization of amplified DNA products were performed by electrophoresis in 1% TopVision Agarose (Fermentas, Lithuania) and a Gel Doc™ XR + device, respectively. Each lane of the agarose gel was loaded with 7 µl of the PCR mixture. Electrophoresis was run in tris–acetate buffer (40 mM Tris–acetate, pH 8.0; 1 mM EDTA) at 5 Volt/cm for 1 h. After electrophoresis, the amplification products were labeled by ethidium bromide and visualized in UV light using a Gel Doc™ XR + device.

HspA I cleaved unmethylated 5′-GCGC-3′ DNA sequences, 5′-GCGC-3′ methylcytosine-containing regions remained unhydrolyzed; thus, unmethylated DNA underwent complete hydrolysis. In such samples, PCR product was not detected in the agarose gel.

Non-hydrolyzed DNA was used as a control to confirm that the absence of amplification product when hydrolyzed DNA was used was the result of DNA bisulfite modification and not an erroneous PCR run or poor quality of the DNA template. Amplification of the Beta-globin gene fragment served as an internal DNA quality control.

Methylation frequency was characterized by the proportion of samples where ADIPOQ gene promoter methylation was detected. Then the frequencies of promoter methylation were compared between groups.

### AdipoR1 and AdipoR2 expression in PBMCs

The AdipoR1 and AdipoR2 were measured in whole PBMC lysate using ELISA Assay Kits (cat.no: MBS2701389 MBS764392 correspondingly, MyBioSource, USA) according to the manufacturer’s protocol. Briefly, the whole-cell lysate was prepared using RIPA buffer. The lysate was cleared from debris by centrifugation for 10 min at 10,000×*g*. Cell lysates and standards were added to the wells of plates pre-coated with capture antibodies and incubated at 370C for 90 min. Afterward, the plates were washed three times, then incubated with a detection antibody at 370C for 60 min. HRP-streptavidin antibodies solution was added to the wells for 30 min at 370C. After washing, TMB substrate was added to each well and incubated for 10 min in the dark, then added a stop solution to each well. Optical density was measured using Microplate reader Synergy H1 at 450 nm. Target concentrations of samples were interpolated from the standard curve. Concentrations of AdipoR1 and AdiporR2 in samples were normalized to total protein.

### Statistical analysis

The results for quantitative variables are expressed as the median and interquartile range (IQR). Qualitative data were compared by the Chi-squared test. Fischer's exact test was used when there was one or more cell(s) with expected values < 5. Wilcoxon sum-rank test was used to compare medians between study groups, and correlation coefficients were computed using the Spearman method. Statistical significance was defined as a value of *p* < 0.05. Standard statistical software was used to conduct the analyses. (Stata 16 and RStudio).

## Results

### Baseline characteristics of the study groups

The demographic and biochemical profile of the 248 participants is listed in Table 2. None of the AD patients had any family history of the disease. Statistical analysis has not revealed significant differences between AD patients and normal controls in terms of ethnicity, age, and gender; however, we have found significantly lower body-mass index (BMI) in the AD group (*p* < 0.001) compared to healthy people. As expected, mini-mental state examination (MMSE) scores were lower in the AD group (*p* < 0.001). The median score of MMSE in AD patients was 14.5 (6–21), implying the disease’s advanced stage. Carriers of ApoE ɛ4 were more frequent in the AD group; 46.9% of AD patients had at least one copy of APOE ɛ 4 (versus 24% in the controls, *p* = 0.002). Except for serum fasting glucose, which was statistically higher in the AD group (*p* = 0.007), and serum triglyceride levels, which were elevated in the control group (*p* = 0.003), other serum biochemical parameters were within the reference range and not significantly different in the two groups.

**Table 2:** Baseline characteristics of the study groups

	AD (n = 98)	Control (n = 150)	*p* value
Age (years)	68 (63–74)	67 (61–74)	0.412
Gender, n (%) *Male* *Female*	29 (29.6%) 69 (70.4%)	58 (38.7%) 92 (61.3%)	0.143
Ethnicity, n (%) *Kazakh* *Russian* *Other*	72 (74.2%) 15 (15.5%) 10 (10.3%)	117 (78%) 24 (16%) 9 (6%)	0.475
Apo e4 carrier, n (%)	46 (46.9%)	36 (24%)	0.002
BMI, kg/m^2^	23.5 (21.9–26.7)	27.4 (24.8–29.8)	< 0.001
MMSE score	14.5 (6–21)	29 (28–30)	< 0.001
Total cholesterol, mmol/L	4.73 (3.79–5.5)	4.63 (3.97–5.36)	0.948
HDL-C, mmol/L	1.2 (0.96–1.5)	1.27 (1.01–1.49)	0.553
LDL-C, mmol/L	3.22 (2.53–3.94)	2.83 (2.4–3.61)	0.059
Triglycerides, mmol/L	1.07 (0.79–1.42)	1.34 (0.91–1.92)	0.003
Fasting glucose	4.96 (4.35–5.72)	4.45 (3.94–5.18)	0.007
Adiponectin (μg/ml)	19.1 (9.9–30.7)	6.2 (2.9–12.7)	< 0.001

The data is presented in medians (IQR) for continuous and as n (%) for categorical variables

### Evaluation of the circulating blood serum adiponectin levels in patients with AD and control group

**Fig. 1:**
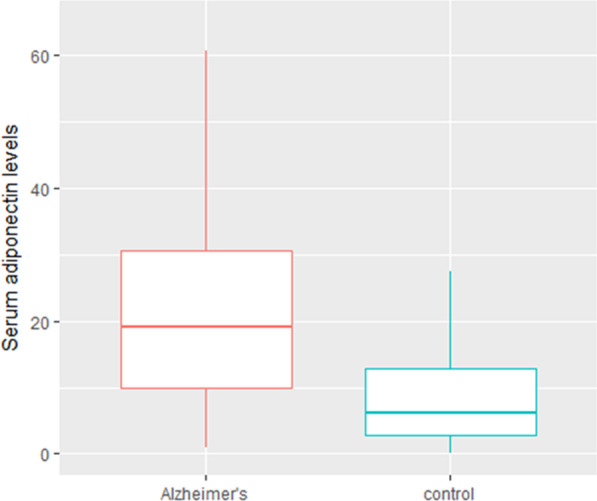
Serum adiponectin levels among control and AD subjects. Box and Whisker plots represent the median, upper median, and lower median of serum adiponectin levels among control and AD subjects (*p* < 0.001)

**Fig. 2:**
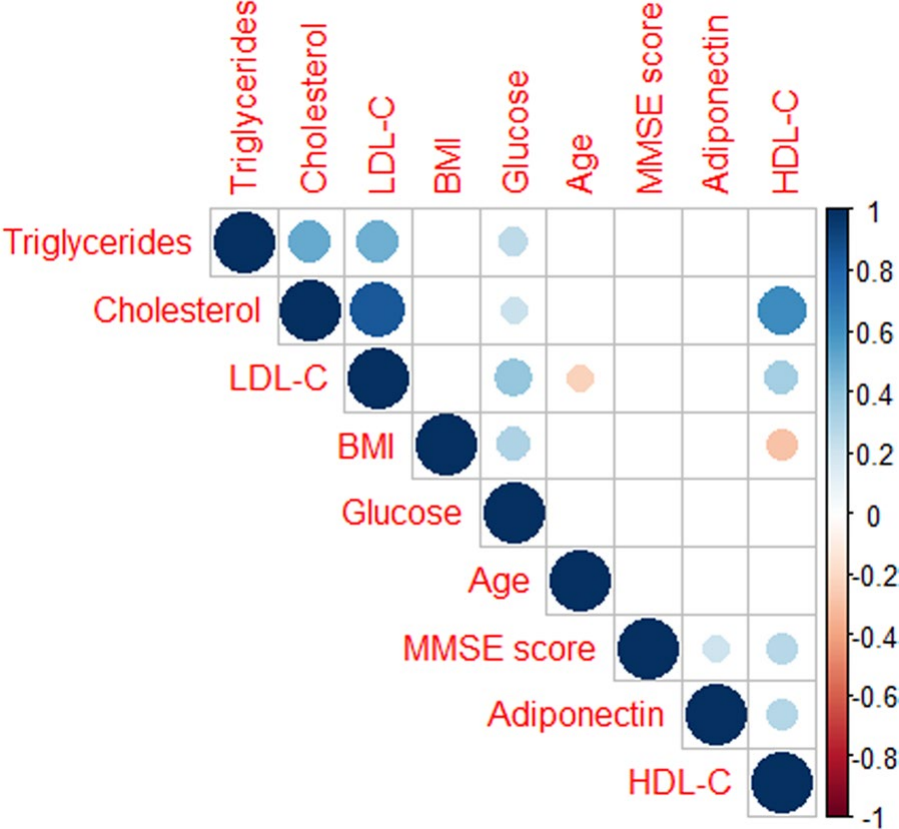
Heat map showing Spearman’s correlations between serum levels of adiponectin and total cholesterol, triglycerides, LDL-C, HDL-C, fasting glucose, BMI and MMSE in AD patients (dark blue—self-self correlations; significant correlations are colored either in light blue (positive) or orange (negative) hues, while non-significant correlations are not shown)

Analysis of adiponectin levels revealed a threefold increase in participants with AD compared to the control group (19.1 vs. 6.2 μg/ml respectively, *p* < 0.001) (Table 2, Fig. 1). We did not find correlations between serum adiponectin levels and fasting glucose, total cholesterol, triglycerides, low-density lipoprotein cholesterol (LDL-C), participants’ ages, and BMI. However, we have found a positive correlation between adiponectin and MMSE scores (r = 0.2, *p* < 0.05) and high-density lipoprotein cholesterol (HDL-C) (r = 0.3, *p* < 0.05) in AD patients (Fig. 2).

### Evaluation of the methylation of adiponectin gene promoter in patients with AD and control group in association with plasma adiponectin levels

**Fig. 3:**
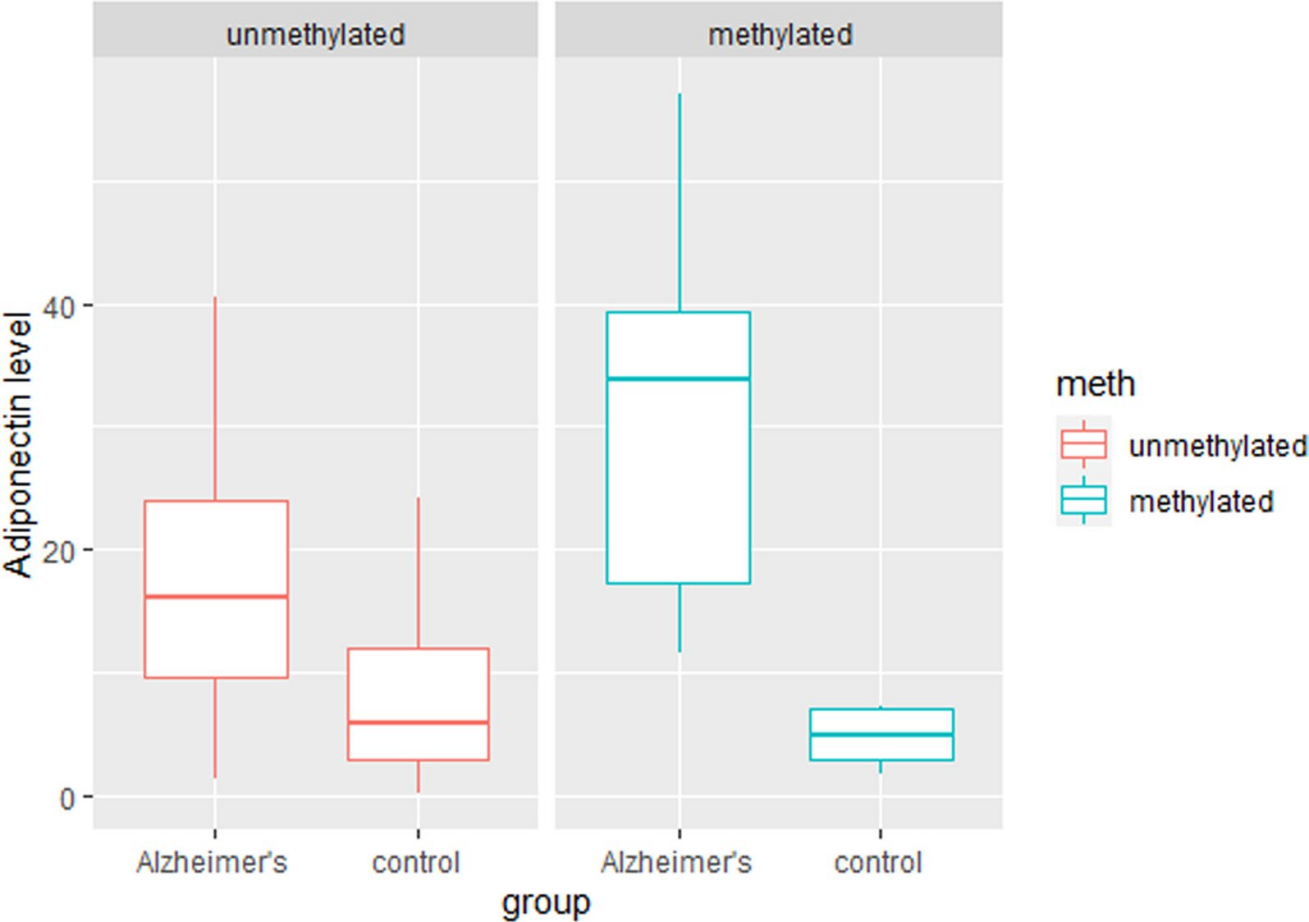
Serum adiponectin levels in AD and control groups by methylation status. Box and Whisker plots represent the median, upper median, and lower median of serum adiponectin levels between methylated and non-methylated samples

Methylation frequency was measured in 166 participants (Table 3). When patients were compared to controls, a substantial difference in the proportion of methylation of CpG sites at 74 nt of the ADIPOQ gene promoter was discovered. (*p* = 0.00643). The methylation frequency at site − 74 nt was 20.97% in AD patients and 5.77% in the control group. We have also observed that serum adiponectin level was significantly higher in methylated samples in the AD group compared to controls (*p* = 0.012). In contrast, in non-methylated samples, we did not observe significant differences in serum adiponectin levels between study groups (Fig. 3).

**Table 3:** Methylation frequency of the ADIPOQ promoter at − 74 nt in AD patients and control groups

	AD N = 62		Controls N = 104	
	N	%	N	%
Unmethylated	49	79.03	98	94.23
Methylated	13	20.97*	6	5.77*

*Chi-square test was used to test proportion difference (*p* = 0.006)

### Evaluation of AdipoR1 and AdipoR2 levels in in PBMCs isolated from the blood of the patients with AD and control group

**Fig. 4:**
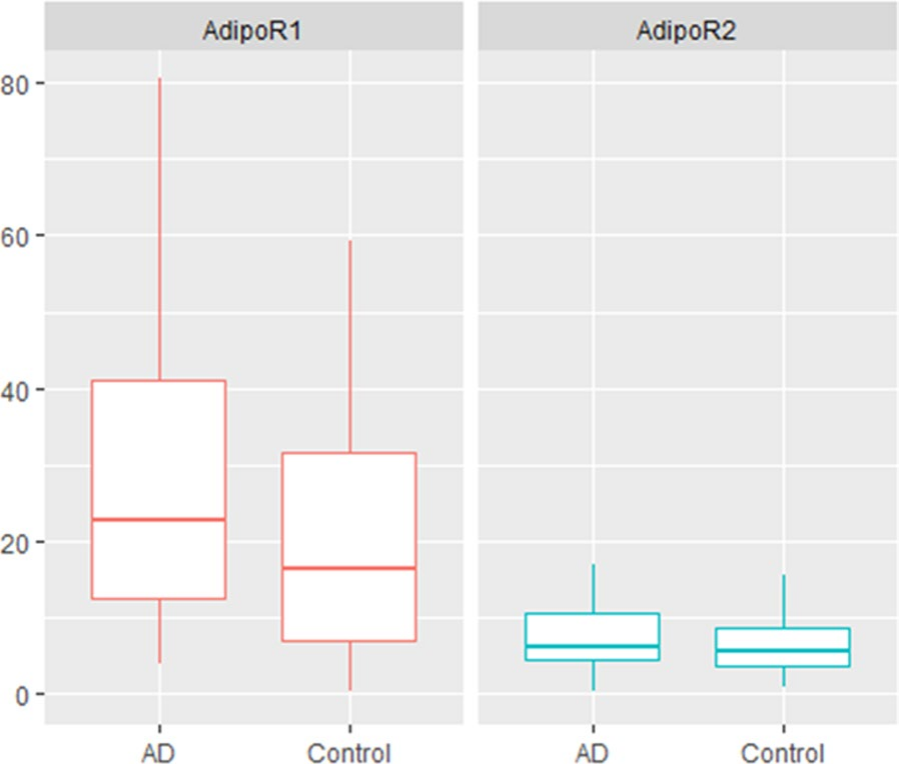
AdipoR1 and AdipoR2 levels in whole PBMC cell lysate. Concentrations in samples were normalized to total protein (ng/mg of total protein). Box and Whisker plots represent the median, upper median, and lower median of AdipoR1 and AdipoR2 levels among control and AD subjects

To identify how the expression of adiponectin receptors were altered in patients with AD, ELISA was performed on human PBMS samples. The median concentrations of AdipoR1 and AdipoR2 were 22.8 (12.6–40.9) and 6.17 (4.68–10.6) ng/mg of total protein in the group of AD patients, and 16.6 (7.15–31.5) and 5.61 (3.78–8.75) ng/mg of total protein in the controls, respectively. We have found that the amount of AdipoR1 was significantly higher among AD subjects (p = 0.04), and the expression of AdipoR2 did not vary between AD patients and controls (*p* = 0.234) (Fig. 4).

## Discussion

In the present study, we have investigated circulating adiponectin levels, adiponectin receptors, and methylation of adiponectin gene promoter in relation to Alzheimer’s disease. As mentioned, the association between adiponectin and AD is disputed; where some studies suggest that lower levels of circulating adiponectin have a neuroprotective role [17], while some studies report an increased risk of AD [40]. Furthermore, one recent prospective population-based cohort study revealed no relationship between serum adiponectin levels and the risk of dementia [41]. In our study, we have observed an elevated level of adiponectin in the blood serum of AD subjects compared to control, in line with the studies in which higher serum adiponectin levels were significantly associated with cognitive deficiency [42], MCI, and AD [12, 14, 40, 43, 44]. Moreover, we have found a positive correlation of serum adiponectin with MMSE scores in AD patients; however, at the moment, further studies are needed to investigate if this correlation is a result of a causal relationship. In this regard, there are two contradictory hypotheses: either elevated adiponectin is harmful, increasing the risk of developing cognitive impairment, or this hormone increases in people who, despite the asymptomatic state, already have neuropathological changes in AD, and adiponectin is necessary for battling neurodegeneration. Increased serum ADIPOQ level might be a result of adiponectin resistance in response to disease progression due to decreased cell signaling efficacy and/or progressive compensatory response to underlying neuro-vascular inflammation [45].

In our study, AD subjects had higher serum adiponectin and lowered BMI than controls at the time of the measurements. Since some studies report that serum levels of adiponectin are dependent on body adiposity [46] and related to obesity-associated cognitive decline [47], it might have substantial consequences on disease pathogenesis. However, we do not have information on their midlife weight, particularly during midlife; thus, it poses certain limitations to drawing a reliable conclusion in our study regarding associations of adiponectin, BMI, and risks of dementia. We have also found that serum adiponectin levels were positively correlated with HDL-C. This observation is in line with the fact that adiponectin plays a considerable role in HDL metabolism [45], and a robust correlation exists between circulating levels of plasma adiponectin and HDL-C [48, 49].

Although the physiological role of ADIPOQ has been intensively investigated, the precise mechanisms regulating the expression of the ADIPOQ gene are unknown [50, 51]. Several studies demonstrated that transcriptional regulation of the human ADIPOQ promoter region is a complex process that involves multiple regulatory elements [51] and binding sites for PPARG2, LRH, RXR, CEBPA, SREBP1c, TFAP2B, FOXO1, SP1, SIRT1, NCOR1, and NCOR2 have been identified in the adiponectin gene promoter region [52]. As mentioned, one of the key regions of the adiponectin gene promoter associated with the transcriptional regulation of adiponectin expression is the E-box [33, 34]. For instance, the circadian rhythm of adiponectin synthesis in mice has been shown to be associated with the transcription factor BMAL1 (Brain and Muscle ARNT-Like 1), which binds directly to E-boxes of the adiponectin gene [53]. Interestingly, epigenetic modifications of one of the E-boxes (CpG sites at − 74) of the adiponectin gene have been associated with metabolic disorders in adolescents [33]. The authors demonstrated a connection between obesity and the methylation status of the CpG site at 74 nt in the ADIPOQ gene promoter, where a decline in the proportion of obese and morbidly obese patients who had this CpG site methylated and the similar proportion of patients in the insulin- and non-insulin-resistant groups who had this CpG site methylated was observed. In our study, we have demonstrated that the methylation frequency of the ADIPOQ gene promoter at CpG sites at − 74 was increased by more than 3.6 times in AD patients and was directly related to serum adiponectin level. Given the relationship between BMI, obesity, adiponectin, and Alzheimer's disease [54–56], we speculate that the methylation of the CpG site at − 74 nt of the ADIPOQ gene promoter in peripheral blood is associated with Alzheimer’s disease.

However, our observation is at variance with the notion that methylation is generally associated with transcriptional silencing [57]. The difference in DNA methylation patterns between adipose tissue and blood cells reported by Ott et al. [31] might be the possible factor explaining the discrepancy between our observations and current ideas about methylation and adiponectin expression. On the other hand, recent research demonstrated that increased methylation of ADIPOQ gene promoters was associated with elevated adiponectin expression in HIV-positive females [58]. These authors also suggest that the ADIPOQ mRNA expression regulation is a complex process, and DNA methylation may even induce gene expression [58]. Interestingly, Houshmand-Oeregaard et al. revealed decreased ADIPOQ gene expression accompanied by elevated ADIPOQ DNA methylation in subcutaneous adipose tissue (SAT) but no correlation between plasma levels of adiponectin and methylation of ADIPOQ gene in SAT in children born from women with gestational diabetes [59]. These findings are compatible with other studies [60] showing a relatively weak relationship between the secretion of adiponectin and the serum adiponectin level. Hoffstedt et al. have linked this observation to the slow rate of turnover of adiponectin (plasma half-life of adiponectin is about 2.5 h) and low rate of adiponectin production [60].

As mentioned, ADIPOQ binds to 3 receptors (AdipoRs): adiponectin receptor type 1 and type 2 (G protein-coupled receptors) and T-cadherin (cadherin family) [5, 61, 62]. AdipoR1 is primarily associated with adiponectin’s metabolic functions, while AdipoR2 is also engaged in the anti-oxidative and anti-inflammatory actions of ADIPOQ [45]. There is data indicating direct associations between plasma ADIPOQ levels and the expression of adiponectin receptors in peripheral blood mononuclear cells (PBMC) [5, 9, 22, 29, 30]. For example, Kollias et al. have reported decreased plasma adiponectin levels and reduced AdipoR1 and AdipoR2 expression in monocytes from overweight coronary artery disease (CAD) patients [9]. Additionally, Luo et al. demonstrated enhanced adiponectin actions by overexpression of AdipoR1 in macrophages [29], while suppression of ADIPOR1 promoted memory dysfunction and the development of AD-like pathology in ADIPOR1 knockout mice [63]. In our study, we observed increased levels of ADIPOQ in plasma and AdipoR1 in PBMCs isolated from AD patients compared to control subjects, while the expression of AdipoR2 did not vary between controls and AD patients.

In fact, a dysfunction of the macrophage system is observed in AD patients, in particular, the impaired ability of Aβ phagocytosis [64–67]. It is an interesting fact that adiponectin regulates the M1/M2 polarization phenotype of peripheral macrophages [30, 68], and this polarization plays a role in the regulation of Aβ oligomers phagocytosis in AD [69, 70]. Reversibly, macrophage polarization phenotype regulates AdipoRs expression and adiponectin anti-inflammatory response [30]. In M1 macrophages, ADIPOQ stimulates pro-inflammatory cytokines TNF-ά, IL-6, and IL-12 and increases AdipoRs expression, while in M2 macrophages, ADIPOQ induces the release of anti-inflammatory cytokine IL-10 and does not affect AdipoRs [30]. These findings allowed us to suggest that altered regulation of plasma ADIPOQ levels and expression of AdipoRs in peripheral monocytes might be one of the factors leading to the dysfunction of the macrophage system, including the impaired ability of Aβ phagocytosis and neuroinflammation in people suffering from AD.

## Conclusion

Our findings revealed an increase in plasma adiponectin levels, enhanced methylation of the adiponectin gene promoter in peripheral blood, and elevated levels of AdipoR1 in PBMCs in persons with Alzheimer's disease. These findings may contribute to a deeper understanding of the factors leading to the development of dementia and may serve as a basis for the advancement of prognostic biomarkers of AD. Nevertheless, longitudinal studies are needed to ascertain our findings.

## Data Availability

The datasets used and/or analyzed during the current study are available from the corresponding author upon reasonable request.

## Abbreviations

ADIPOQ: Adiponectin
NINCDS-ADRDA: National Institute of Neurological and Communicative Disorders and Stroke–Alzheimer’s Disease and Related Disorders
PCR: Polymerase chain reaction
IQR: Interquartile range
BMI: Body mass index
MMSE: Mini-mental state examination
HDL-C: High-density lipoprotein cholesterol
LDL-C: Low-density lipoprotein cholesterol
E-boxes: Enhancer boxes
CpG: Cytosine-phosphate-guanine
